# Experimental Studies on Zooplankton-Toxic Cyanobacteria Interactions: A Review

**DOI:** 10.3390/toxics11020176

**Published:** 2023-02-14

**Authors:** S. Nandini, S. S. S. Sarma

**Affiliations:** Laboratory of Aquatic Zoology, Division of Research and Postgraduate Studies, Universidad Nacional Autónoma de México, FES-Iztacala, Tlalnepantla 54090, State of Mexico, Mexico

**Keywords:** cladocerans, rotifers, copepods, cyanotoxins, bioaccumulation, acute and chronic toxicity tests

## Abstract

Cyanobacterial blooms have been recognized as a problem in fresh water for about 150 years. Over the past 50 years, experimental studies on the subject have gained importance considering the increasing need to control toxic cyanobacterial blooms. This article presents information on the different lines of research that have been undertaken on zooplankton–cyanobacteria interactions over the past 50 years. These include information on filtering/ingestion rates and phytoplankton preferences of small and large rotifers, cladocerans, and copepods; growth rates of zooplankton on cyanobacterial diets; feeding rates of other freshwater invertebrates on cyanobacteria; role of zooplankton in top-down biomanipulation efforts; effect of cyanotoxins on zooplankton; bioaccumulation of cyanotoxins; and physical and chemical control of cyanobacterial blooms. We also highlight measures that have led to successful lake management and improvement of water quality in selected waterbodies.

## 1. Macroanalysis of Works Involving Toxic Cyanobacteria and Zooplankton

**Figure 1:**
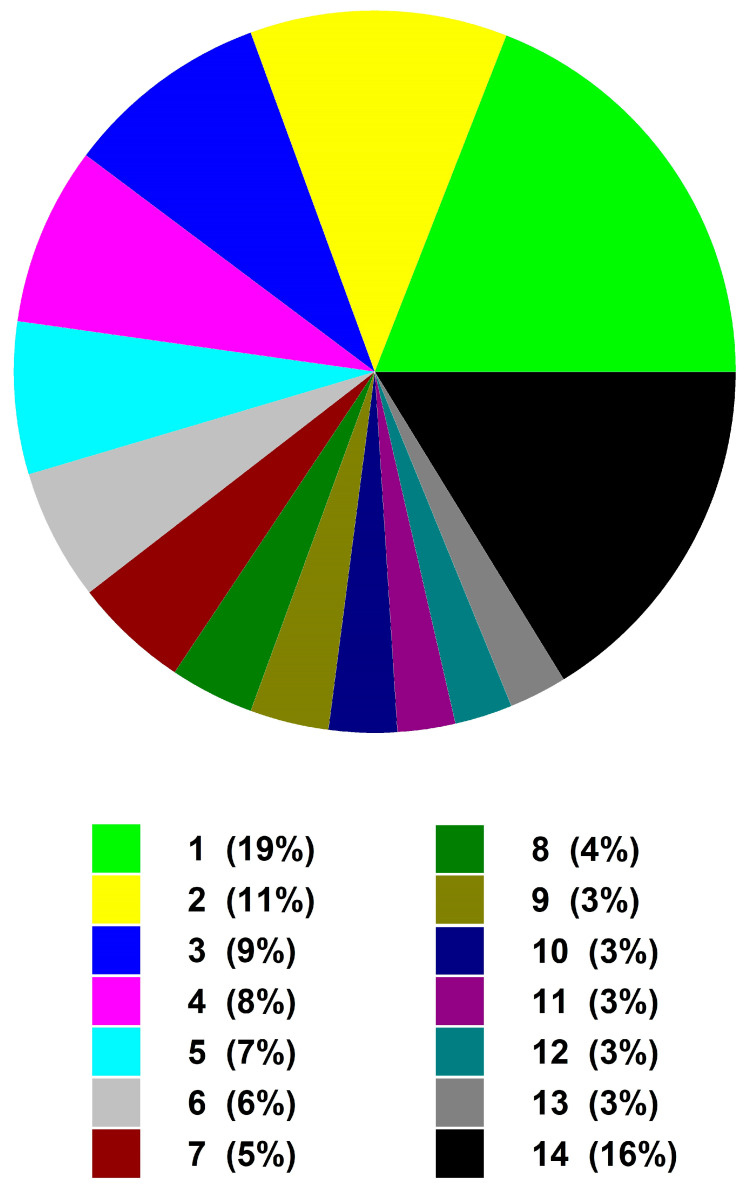
Different fields of research (%) on the zooplankton–cyanobacterial interactions. (1) Cyanobacterial effects on cladocerans and copepods, (2) control of cyanobacteria, (3) limnological effects on cyanobacteria, (4) zooplankton grazing in the field, (5) nutrient effects on cyanobacterial blooms, (6) marine cyanobacterial studies, (7) zooplankton grazing on cyanobacteria and laboratory studies, (8) cyanobacterial consumption by fish, (9) zooplankton vs. cyanobacterial blooms, (10) river studies and grazing, (11) ciliates and cyanobacteria, (12) cyanobacterial effects on rotifers, (13) fish effects on cyanobacteria and zooplankton, and (14) other studies such as molecular studies, paleolimnology, bioaccumulation of cyanotoxins, etc.

Ever since the first report on the toxicity of cyanobacterial blooms by George Francis in 1878, there have been numerous field and laboratory studies on freshwater and marine cyanobacteria. These studies in fresh water are of vital importance considering the limited quantities of this resource and the difficulty in reducing the concentrations of cyanotoxins with water treatment procedures. Bioaccumulation of cyanotoxins in marine and freshwater organisms often results in health risks to humans, another reason for the increased effort in studying the effects of cyanotoxins. Studies on cyanobacterial interactions have focused mostly on those with viruses, bacteria, rotifers, cladocerans, copepods, mollusks, and fish. Based on a data search in the *Web of Science* using the words Cyanobacteria and Zooplankton and Experiment, we retrieved 425 articles. We found that many studies focused on field and laboratory grazing experiments using zooplankton, nutrient effects on cyanobacteria, and limnetic and marine cyanobacteria. However, more than 19% of the studies focused on the effect of cyanobacteria and their secondary metabolites on the fitness of rotifers, cladocerans, and copepods, while more than 10% of the studies were on methods to control cyanobacterial blooms (Figure 1).

Close to 2700 species of cyanobacteria from about 150 genera have been described, and it is predicted that this number will rise to above 6000 in the future [1]. However, less than 20 genera have been actually tested in experimental studies (Table 1). Since *Spirulina* is not toxic to zooplankton, it was excluded from the present analysis. Among zooplankton, most studies focus on the effects of cyanobacteria on rotifers, cladocerans, and copepods. More than 30 species of cladocerans and 25 species of calanoid copepods have been used in experimental studies to test the toxic effects of cyanobacteria. Fewer species (17) of rotifers, cyclopoid copepods (12), and harpacticoid copepods (5) have been used in similar studies (Table 2). Among cladocerans, the emphasis has been on the genus *Daphnia*: nineteen species have been used in experiments and only thirteen species of other genera and species of Cladocera.

## 2. Ingesting Toxic Cyanobacteria

Recent studies indicate the ability of viruses and bacteria to increase the edibility of cyanobacteria. Agha et al. [2] show that cyanobacteria infected by chytrid fungi are better utilized by *Daphnia galeata*. Protist endoparasites such as *Caullerya mesnili*, however, increase the susceptibility of *Daphnia* to cyanobacterial diets [3]. Gut microbiota is also known to facilitate the consumption and digestion of cyanobacteria by cladocerans [4]. These works are few, and further studies are needed to understand the molecular mechanism in such observations. Data on cladoceran tolerance of cyanobacterial diets in different regions of the world indicate that the same crustacean species tolerant in some geographic regions is not the case for other regions [5]. The rotifer *Brachionus calyciflorus* is tolerant to *Cylindrospermopsis raciborskii* isolated from Brazil and consumed the cyanobacteria [6]. On the other hand, the same rotifer species was more sensitive to the same cyanobacterial species isolated from another region. This has been attributed to clone-related differences of the cyanobacterial species in question [7] and the adaptation of the zooplankton taxa. However, it is also possible that this may be due to differences in the gut flora [4].

Many studies on zooplankton cyanobacteria interactions focus on the ability of zooplankton to control cyanobacterial blooms. In a seminal work, Burns [8] showed that large-sized cladocerans could ingest cyanobacteria better. The study shows the importance of large-sized cladocerans in ingesting phytoplankton > 50 µm. Ever since, numerous studies on the filtering rates of cladocerans and other aquatic organisms have shown that large-sized microcrustaceans (>3000 µm) are quite effective in feeding on cyanobacterial colonies, filaments, and detritus [9,10]. Clearance and ingestion rate studies are short-term experiments that provide ample information on the possibility of success in using selected taxa in consuming a given diet and cyanobacteria control [11]. These have been conducted by direct algal counts or using radioactive markers [12].

## 3. Attempts to Control Cyanobacterial Blooms

Ever since the early studies by Schindler et al. [13], Shapiro et al. [14], and Carpenter et al. [15] on the control of nutrient input, trophic cascade, and biomanipulation to control cyanobacterial blooms, several laboratory experiments have been conducted to determine the most effective zooplankton species for cyanobacterial feeding in selected regions for the control of toxic blooms. Small-sized cladocerans such as *Bosminia longirostris*, *Ceriodaphnia cornuta,* and *Ceriodaphnia dubia* often have moderate growth rates and survivorship on cyanobacterial diets [5,16,17]. However, their ability to effectively control blooms in nature is greatly limited since they cannot graze on large colonial or filamentous cyanobacteria. There is evidence of a relationship between temperature, cyanobacterial length, and the body size of cladocerans in effectively feeding on filamentous phytoplankton [10,18]. Daphniids are often considered the most suitable zooplankton to control cyanobacterial blooms. The large body size and adequate growth rates of *Daphnia magna* on cyanobacterial diets [19] indicate that the species is ideally suited for lake management. This is probably the reason for the emphasis on studies on *Daphnia*; nineteen species of the genus *Daphnia* have been used in experiments to test the effects of cyanobacteria or cyanotoxins on their fitness, while thirteen species of all the other cladocerans have been used in similar studies (Table 2).

## 4. Cladoceran Tolerance to Cyanobacteria

Simple experiments to test for the toxicity of algal blooms suggested by Lampert [20] (comparing the survivorship of starved cladocerans to those fed on cyanobacterial diets) were standard practice until the development of ELISA kits and HPLC techniques to quantify cyanotoxins. However, these ecological experiments have a great application; cladocerans often are quite resistant to cyanotoxins. Some studies indicate that previous exposure to toxic cyanobacteria results in an adaptation to cyanobacterial diets [5,21]. Genetic markers are being studied to indicate clones tolerant to cyanobacteria [22]. Proteomics and the study of zooplankton hormones are future lines of research that will help explain the response of different clones and species of zooplankton to cyanobacteria and cyanotoxins [23,24].

## 5. Studies in Temperate and Tropical Regions on Cyanobacterial Control

Many studies on controlling cyanobacterial blooms have used *Daphnia magna* in temperate countries where the species is naturally distributed. *Daphnia* (often <2000 µm) occurs in tropical countries, too, although they may have been first described from temperate regions (*Daphnia carinata, Daphnia ambigua, Daphnia lumholtzi,* and *Daphnia similis*) [25]. In general, however, *Daphnia* spp. are found at low densities in tropical waters, possibly because of high fish predation pressure throughout the year [26] and their sensitivity to toxic cyanobacteria. For the same reasons, the size structure of zooplankton communities in tropical waters is often in favor of small zooplankton (rotifers, copepods, and small cladocerans) [27]. Common cladoceran genera in tropical and sub-tropical waters include *Diaphanosoma, Moina, Chydorus, Alona*, and *Macrothrix*, all of which measure less than 1000 µm. Population growth and life table experiments indicate that some clones of *Moina* can grow well on cyanobacterial diets, especially at high temperatures (>25 °C) [28,29]. On the other hand, the sensitivity of some clones of *Moina* to cyanobacterial diets is attributed to the adverse effect of these diets on the functioning of digestive enzymes, especially proteases [30].

Dense and persistent cyanobacterial blooms are often found in tropical waters almost throughout the year. Smaller-sized zooplankton present in such waters are not as effective cyanobacteria grazers as large (>3000 µm) daphniids of temperate regions. Hence, the need exists to determine the grazing potential of other crustaceans common in these regions. Ostracods are known to feed on some filamentous cyanobacteria [31]. Some observational field studies and laboratory experiments show that ostracods and amphipods are effective feeders of cyanobacteria [32,33]; their ability to control cyanobacterial blooms on a large scale needs to be tested.

Numerous studies have been conducted on the effect of cyanobacteria or cyanotoxins on zooplankton. Discussing all of them is beyond the scope of this manuscript; hence, some selected studies are highlighted in Table 3. Daphniids are often quite sensitive to cyanobacteria and cyanotoxins, and many species of zooplankton die when fed *Microcystis*. *Daphnia laevis* is an interesting species with regard to its ability to tolerate cyanobacterial blooms in the tropics. Nandini et al. [21] showed that *Daphnia laevis* has higher growth rates on cyanobacterial diets from the reservoir Valle de Bravo than chlorophytes. Recent works show that *Daphnia* has specific enzymes that enable it to tolerate cyanotoxins [34]. *Daphnia laevis* is quite common in lakes and reservoirs with cyanobacteria in Mexico. *Daphnia laevis* is also capable of bioaccumulating cyanotoxins and shows a high degree of tolerance to these compounds, as evident from laboratory [21,29] and field studies [35].

## 6. Zooplankton Tolerance to Toxic Cyanobacteria

Extrapolating laboratory findings to field situations based on studies using a single experimental design is perhaps unwise. For instance, several field and laboratory experiments have been conducted using *Diaphanosoma*, a common cladoceran in cyanobacteria-dominated lakes. Fulton and Paerl [49] showed in laboratory experiments that compared to *Daphnia*, *Diaphanosoma brachyurum* had a greater tolerance to *Microcystis*. While previous exposure to *Microcystis* helps *Daphnia* improve its tolerance to this diet [50], demographic experiments on *Diaphanosoma mongolianum* regarding its tolerance to *Microcystis* diets show that it is high regardless of previous exposure to a cyanobacterial diet [37]. Species-specific differences in the clearance rates of *Diaphanosoma* are also well established based on laboratory studies [51,52]. Pagano [53], in feeding experiments, showed that *Diaphanosoma* does not feed on large food particles but mostly on particles < 4.5 µm to 10 µm. Gut content analyses indicate the presence of *Dolichospermum* in *Diaphanosoma* in reservoirs in Poland [54]. Recent studies also show that some species of *Diaphanosoma* such as *D. celebensis* are capable of microcystin uptake and tolerance [55]; these studies also indicate the importance of molecular markers in biomonitoring of aquatic water bodies. These findings help explain the frequent occurrence of Diaphanosoma in reservoirs dominated by cyanobacteria around the world [37,56].

Maternal exposure to cyanobacteria helps improve fitness although a meta-analysis by Radersma et al. [57] showed that these interactions are weak. We also found similar results in the fitness of two strains of *Diaphanosoma mongolianum* with and without previous exposure to *Microcystis* [37]. Tolerance to cyanotoxins as a consequence of previous exposure has also been reported in the literature. For example, *Daphnia magna* previously feeding on cyanobacteria showed greater tolerance to these diets, as do their offspring [58]. Similar trends have been reported for two clones of *Ceriodaphnia cornuta* fed on *Microcystis aeruginosa*: one isolated from a water body without *Microcystis* and the other from a pond with *M. aeruginosa* [5].

## 7. Rotifers as Grazers on Toxic Cyanobacteria

Rotifers are common in eutrophic tropical and temperate lakes and reach high densities in spite of the presence of cyanobacteria [59,60]. Although feeding experiments and demographic studies on rotifers such as *Brachionus calyciflorus, B. havanaensis, Hexarthra mira, Keratella cochlearis*, and *Synchaeta pectinata*, among others, indicate their ability to feed on cyanobacteria such as *Microcystis aeruginosa, Anabaena flos aquae*, and *Anabaena affinis* [5,61,62], their feeding and clearance rates are not enough to effectively reduce cyanobacterial blooms. Some species such as *Brachionus havanaensis* are common in tropical lakes in the Americas; laboratory experiments also indicate that this rotifer can grow well on unicellular cyanobacteria [59]. The adverse effects of a cyanobacterial diet (*Microcystis, Anabaena*, and *Dolichospermum*) and cyanotoxins (pure and crude extracts) on *Brachionus calyciflorus* have been well documented [5,39,62,63]. Often, the abundant taxa in lakes with dense cyanobacterial blooms include *Keratella* and *Polyarthra*. These are bacterivores, probably avoiding the adverse effects of cyanotoxins since they cannot ingest large colonies or filaments. *Brachionus havanaensis*, which is native to the neotropical region [64], has recently been reported from L’Albufera, Valencia, where it is an exotic species, feeding on cyanobacteria when offered in the sonicated form [60].

## 8. Demographic and Population Level Parameters of Zooplankton Fed Mixed Cyanobacterial-Algal Diets

Species of rotifers and cladocerans when fed on an exclusive diet of cyanobacterial such as *Microcystis* had lower growth rates than those fed on green algae (Table 4). Though various species of toxic cyanobacteria often occur as blooms, they rarely constitute 100% of the phytoplankton. They coexist with species of edible algae, albeit at lower densities [65]. Therefore, zooplankton in nature can exploit the seasonal changes in the proportion of toxic cyanobacteria and edible algal species. During winters, especially in high-altitude waterbodies, cyanobacterial blooms are not common, and the relative proportion of edible algae increases. Under such conditions, rotifers and cladocerans can effectively feed on the edible fraction of phytoplankton or include some portion of toxic cyanobacteria for their growth [66]. Cyanobacterial species also contain some useful energy components in their chemical composition, such as proteins [67]. Therefore, feeding on a mixed diet involving a small proportion of toxic cyanobacteria with a higher proportion of edible algae may offer better growth rates than exclusively relying on limited edible algae. Alva-Martínez et al. [68,69] conducted population growth experiments on selected species of rotifers and cladocerans using different proportions of cyanobacteria with green algae (100–0%). In general, their studies revealed higher population growth rates of zooplankton on mixed diets than algae or cyanobacteria alone. This suggests that in nature, some species of zooplankton show higher population abundances, probably due to their generalist feeding on available seston [11].

In cyanobacteria-dominated waterbodies, certain zooplankton dominate, while others remain in low abundance [78]. However, with seasonal changes, the composition of phytoplankton varies, favoring other zooplankton species. The dominance of one species of zooplankton over others in waterbodies with cyanobacterial blooms is also due to the relative sensitivities of zooplankton. Therefore, the competing abilities of zooplankton species depend on their ability to resist cyanotoxins [79]. This is usually a conjecture if the interpretation is exclusively based on field samples. Laboratory studies on competition involving toxic cyanobacteria as diet confirm the relative competitive abilities of two or more zooplankton species for limited resources. The competition study by Sarma et al. [60] indicates the ability of the exotic *Brachionus havanaensis* to outcompete the native *B. angularis*, especially on *Microcystis* diets; this cyanobacterium forms dense blooms in L’ Albufera. Demographic studies on the effect of extracts from phytoplankton blooms using rotifers provide valuable insights into natural interactions. For instance, Zamora Barrios et al. [38] studied the tolerance of clones of *B. calyciflorus* from Lake Texcoco, a saline lake with dense cyanobacterial blooms. It was clear that the crude extracts were not toxic to *B. calyciflorus*, unlike in the study by Nandini et al. (2019) [43], where extracts from dense blooms of *Woronichinia* in the freshwater reservoir of Valle de Bravo were toxic to *B. calyciflorus*. Kotut and Kreinitz [80] suggested that *Microcystis* from saline lakes are often low in toxicity and may represent cryptic species different from the freshwater *Microcystis aeruginosa*.

## 9. Grazing and Selection

Among the different groups of zooplankton, copepods can selectively feed on edible particles such as algae, detritus, and bacteria while simultaneously avoiding ingestion of toxic cyanobacteria [81]. This conclusion can also be derived based on gut content analysis of copepods and laboratory studies involving analysis of phytoplankton composition before and after addition of copepods in microcosms [11,82]. Copepods also show marked species-specific responses to cyanobacterial diets. Feeding studies indicate that copepods can detect the quality of the diet and often avoid toxic cyanobacteria [81]. This, along with their high swimming speed and the ability to avoid fish predators [83], makes them, with rotifers, the dominant taxa in tropical lakes and reservoirs [84]. However, species-specific responses in the feeding preferences of copepods are frequently reported; for instance, Kâ et al. [85] reported that *Mesocyclops ogunnus* does not feed on cyanobacteria, while *Mesocyclops thermocyclopoides* and *Mesocyclops pehpeiensis* can feed and reproduce on cyanobacterial diets, albeit not as well as on animal diets [86,87]. Xu and Burns [88] also showed that three species of *Boeckella* can grow well on an exclusive diet of *Anabaena oscillarioides*. With the development of molecular tools, a recent study by Gorokhova et al. [22] indicated that the year-long expression of mlrA genes (codes for the production of microcystinase, an enzyme that hydrolyzes microcystins) from the gut of the marine copepods *Acartia bifilosa* and *Eurytemora affinis* allows them to feed effectively on *Nodularia spumigena*. Further studies are needed to explain the mechanisms behind the species-specific responses of copepods to cyanobacterial diets and cyanotoxins.

The ability of cladocerans and rotifers to selectively avoid ingestion of toxic cyanobacterial cells can be easily confirmed from laboratory studies. In most mixed-diet (cyanobacteria and edible algae, mentioned earlier) studies, the quantity of unconsumed cyanobacterial cells is rarely quantified, and therefore, it is difficult to derive the energy budget of such studies [68]. Carefully executed short-term grazing studies certainly offer some light on the differential feeding rates from algal-cyanobacterial mixed diets. This approach becomes much easier if the diet composition can be identified based on their morphological differences. To avoid mechanical impediments, large colonies of cyanobacteria such as *Microcystis* spp. are sonicated to single cells and mixed with single-celled non-colonial algal species such as *Scenedesmus*. The mixed diet can be offered in different proportions to the grazers, and after a specific period of feeding, the unconsumed cells of cyanobacteria and alga can be quantified. Pérez-Morales et al. [89] showed that although the cladocerans *Daphnia pulex, Moina micrura,* and *Ceriodaphnia dubia* consumed sonicated cells of toxic *Microcystis aeruginosa*, when mixed with algae, the consumption of cyanobacterial cells varied depending on the proportion of the mixed diets. Thus, when the algal cell density in the mixed diet was higher, the rate of consumption of *M. aeruginosa* cell numbers by *D. pulex* and *M. micrura* was lower. The same study using rotifers (*B. calyciflorus*, *B. rubens*, and *Plationus patulus*) showed differential feeding rates on cyanobacteria in mixed diets. Thus, the study showed that the consumption of cyanobacteria by the zooplankton also depends on the availability and proportion of the edible fraction of seston in natural water bodies.

## 10. Effects of Crude and Purified Extracts from Toxic Cyanobacteria

Even after the collapse of cyanobacterial blooms, water toxicity exists in lakes and reservoirs. This is due to the lysis of cyanobacterial cells and the release of cyanotoxins into the medium. Estimating the concentration of and effects of cyanotoxins in reservoir water should be routine in water management protocols [90]. Evaluating the effects of pure cyanotoxins and crude extracts from blooms in lakes and reservoirs on zooplankton is on the rise over the past couple of decades. The latter are easily obtained using cycles of freezing, thawing, and sonication of concentrated blooms from natural waterbodies [91]. The effect of these extracts on locally available zooplankton taxa makes it easier to extrapolate the findings from laboratory studies to nature. The adverse effects of cyanobacterial extracts have been studied since the early 2000s [30,92] on several cladocerans including *Moina macrocopa*, *Ceriodaphnia dubia*, *Ceriodaphnia silvestrii,* and *Daphnia similis*. Ever since, many studies indicate the toxicity of extracts from blooms on rotifers and cladocerans [29,38,39,41,43,93]. These studies are based on acute and chronic toxicity tests and data related to survivorship and fecundity of the test zooplankton. Some species of cyanobacteria, *Woronichinia*, for instance, are difficult to culture under laboratory conditions [94]; extraction of cyanotoxins from field-collected blooms enables assessment of their toxicity to zooplankton [36]. Other effective short-term experiments not frequently used to analyze the effect of cyanobacteria on cladocerans are the heartrate measurements when exposed to toxins. Recently, Araiza Vazquez et al. (in prep) showed that there was approximately a 30% decline in the number of palpitations of the heart of *Simocephalus vetulus* when exposed to cyanotoxins from a bloom of *Microcystis aeruginosa* in the Virgilio Uribe rowing canal in Mexico City. This simple and effective experimental design yields quantitative results on the toxicity of water with cyanobacterial blooms.

## 11. Bioaccumulation of Cyanotoxins through the Food Chain

The bioaccumulation of cyanotoxins in zooplankton has been well demonstrated. Early studies by Watanabe et al. [95] indicated high levels of bioaccumulation in small cladocerans such as *Bosmina* and *Diaphanosoma* when exposed to *Microcystis* diets. With the advent of ELISA, more reliable methods also indicated the accumulation of cyanotoxins in generalist filter feeders, mostly cladocerans [12]. The degree of bioaccumulation of cyanotoxins can be related to the feeding rates and filtering behavior of the species; [96]. Zamora Barrios et al. [38] suggested that the low bioaccumulation of cyanotoxins in cladocerans such as *Bosmina* in a Mexican lake is due to their inability to ingest the dominant cyanobacterial taxa, which are present as large filaments or colonies (*Planktothrix, Anabaena, Microcystis*, and *Cylindrospermopsis*). Some cladocerans such as *Diaphanosoma* and *Daphnia laevis* are more tolerant to cyanotoxins in their tissues than other species [55,97,98].

## 12. Lake Management

An important reason for the increased interest in cyanobacterial research is the global presence of persistent cyanobacterial blooms in freshwaters. Several excellent reviews [99] and books [100] have been written on the subject in recent years. Physical, chemical, and biological methods to control cyanobacteria are being attempted but with limited success [99]. A holistic approach involving bottom-up and top-down control [101] will yield satisfactory results.

## 13. Conclusions

Many studies have considered the adverse effects of cyanobacteria on zooplankton based on both field and laboratory studies. These studies offer some generalizations on how toxic cyanobacteria shape the zooplankton community structure under field conditions and changes in the demographic parameters of rotifers, cladocerans, and copepods under laboratory conditions. Short-term feeding and mesocosm studies show how effective grazing by zooplankton is in controlling toxic cyanobacterial blooms. Most studies suggest that large cladocerans, particularly daphniids, can reduce toxic cyanobacterial blooms. Previous exposure to cyanotoxins is known to increase the tolerance of zooplankton to cyanobacteria. Laboratory experiments on the demographic responses of zooplankton to cyanobacterial diets and cyanotoxins permit the explanation of the dominance of these taxa in nature. Regarding toxicity tests, a given zooplankton species may respond differently when exposed to the same cyanobacterium. These differences are mainly due to difficulties in the identification of cyanobacteria (lack of molecular approach in the taxonomic determination [80]), differences in cyanobacteria and zooplankton, loss of cyanotoxicity in long-term laboratory cultures with relation to nitrogen availability [102], previous exposure to toxic cyanobacteria, multi- and trans-generational adaptation of zooplankton, bioassay design, and tests conducted under field or laboratory conditions. Studies aimed at reducing toxic cyanobacterial blooms using crustaceans (other than cladocerans and copepods) such as ostracods and amphipods are few. Other modes of control (chemical or physical agents) such as by nutrient-based approach or intra-primary producer interactions (e.g., allelopathic effects) have not been considered here, as they are beyond the scope of this work. An integrated approach involving different methods is much needed to reduce the cyanobacterial blooms in ponds, lakes, and reservoirs.

## Figures and Tables

**Table 1:** List of cyanobacterial genera used to evaluate their effects on zooplankton.

Order: Chroococcales
*Aphanothece*
*Microcystis*
*Woronichinia*
Order: Nostocales
*Anabaena*
*Aphanizomenon*
*Cylindrospermopsis*
*Cylindrospermum*
*Microcoleus*
*Nodularia*
*Scytonema*
*Spirulina*
Order: Oscillatoriales
*Arthrospira*
*Lyngbya*
*Phormidium*
*Planktothrix*
*Pseudanabaena*

**Table 2:** Species of major zooplankton groups (Rotifera, Cladocera, and Copepoda) used in experimental studies with cyanobacteria.

*Group/Species*
**Rotifera**
*Asplanchna girodi*
*Brachionus angularis*
*Brachionus calyciflorus*
*Brachionus dimidiatus*
*Brachionus falcatus*
*Brachionus havanaensis*
*Brachionus plicatilis*
*Brachionus rubens*
*Brachionus urceolaris*
*Euchlanis dilatata*
*Hexarthra mira*
*Keratella cochlearis*
*Keratella testudo*
*Lecane inermis*
*Plationus patulus*
*Polyarthra vulgaris*
*Synchaeta pectinata*
**Cladocera**
*Bosmina fatalis*
*Bosmina longirostris*
*Ceriodaphnia cornuta*
*Ceriodaphnia dubia*
*Ceriodaphnia rigaudi*
*Daphnia ambigua*
*Daphnia carinata*
*Daphnia cucullata*
*Daphnia galeata*
*Daphnia gessneri*
*Daphnia hyalina*
*Daphnia laevis*
*Daphnia longispina*
*Daphnia lumholtzi*
*Daphnia magna*
*Daphnia mendotae*
*Daphnia obtusa*
*Daphnia parvula*
*Daphnia pulex*
*Daphnia pulicaria*
*Daphnia retrocurva*
*Daphnia similis*
*Daphnia similoides*
*Daphnia sinensis*
*Diaphanosoma brachyurum*
*Diaphanosoma celebensis*
*Diaphanosoma mongolianum*
*Macrothrix spinosa*
*Moina macrocopa*
*Moina micrura*
*Scapholeberis kingii*
*Simocephalus vetulus*
**Copepoda**
**Calanoids**
*Acartia bifflosa*
*Acartia clausi*
*Acartia lilljeborgii*
*Acartia tonsa*
*Acrocalanus gibber*
*Acrocalanus gracilis*
*Arctodiaptomus dorsalis*
*Boeckella dilatata*
*Boeckella hamata*
*Boeckella propinqua*
*Boeckella triarticulata*
*Calanus finmarchicus*
*Diaptomus birgei*
*Diaptomus floridanus*
*Eudiaptomus gracilis*
*Eurytemora affinis*
*Eurytemora carolleeae*
*Notodiaptomus iheringi*
*Paracalanus parvus*
*Pseudodiaptomus forbesi*
*Pseudodiaptomus hessei*
*Pseudodiaptomus inopinus*
*Sinocalanus tenellus*
*Temora longicornis*
*Temora stylifera*
*Temora turbinata*
**Cyclopoids**
*Acanthocyclops robustus*
*Cyclops kolensis*
*Cyclops vernalis*
*Cyclops vicinus*
*Diacyclops thomasi*
*Mesocyclops leuckarti*
*Mesocyclops ogunnus*
*Mesocyclops pehpeiensis*
*Mesocyclops thermocyclopoides*
*Metacyclops mendocinus*
*Thermocyclops decipiens*
*Thermocyclops oithonoides*
**Harpacticoids**
*Attheyella trispinosa*
*Canuella perplexa*
*Euterpe acutifrons*
*Macrosetella gracilis*
*Tigriopus japonicus*

**Table 3:** Selected data on the effects of cyanobacteria/cyanotoxins on zooplankton based on laboratory and field studies.

Authors	Toxins/Concentrations Tested	Waterbody	Cyanobacteria	Zooplankton
Nandini et al. 2017 [36]	Concentration of microcystins in the extract (see methods) obtained during the bloom was 138 µg L^−1^; sestonic microcystins in Lake L’Albufera 0.8 µg to 1.94 µg L^−1^	Lake L’Albufera, Spain	*Microcystis* spp.	*Moina micrura*
Nandini et al. 2021 [37]	Median lethal concentration on *Moina macrocopa* was 1.56, 1.30, and 2.56 µg L^−1^ in June, September, and March, respectively.	Virgilio Uribe, Mexico	*Microcystis* spp.	*Moina macrocopa*
Zamora Barrios et al. 2017 [38]	0.20 and 2.4 µg L^−1^ in the lake water.	Lake Texcoco, Mexico	*Planktothrix, Anabaenopsis, Spirulina,* and *Microcystis*	*Brachionus calyciflorus*
Zamora Barrios et al. 2015 [39]	1.504 µg L^−1^	Valle de Bravo, Mexico		*Ceriodaphnia cornuta; Plationus patulus*
Freitas et al. 2014 [40]	48 h LC_50_ values of cyanobacteria: hepatotoxic (ranging from 216.69 to 270.25 mg L^−1^) and neurotoxic (ranging from 210.74 to 234.74 mg L^−1^)	Jacarepaguá Lagoon, Rio de Janeiro, Brazil	*Anabaena spiroides*; *Microcystis aeruginosa*	*Daphnia magna*
Nandini et al. 2020 [41]	0.14–10.8 µg L^−1^	Chapultepec Lake, Mexico	*Microcystis* spp.	*Brachionus havanaensi, Brachionus calyciflorus*, *Moina macrocopa*
Vo et al. 2020 [42]	The 24 h and 48 h LC_50_ values for MC-LR ranged from 247–299 and 331–409 µg MCE L^−1^, respectively.		*Microcystis aeruginosa*; *Cylindrospermopsis curvispora*	*Daphnia lumholtzi*
Nandini et al. 2019 [43]	Microcystin concentration in lake water was 9.57 µg L^−1^ and 0.097 µg L^−1^; LC_50_ was 5.34 and 0.035 µg microcystin L^−1^ in January and September, respectively.	Valle de Bravo, Mexico	*Microcystis flos aquae* (January); *Woronichinia naegliana* (September)	*Brachionus calyciflorus*
Jungmann and Benndorf1998 [44]	1.6 to 4.3 µg mg^−1^ dry weight. LC_50_ of 36 µg ml^−1^ dry weight of *Microcystis* and hepatotoxic microcystins are different from DTC (*Daphnia*-toxic compound)	Bautzen Reservoir, Germany	*Microcystis* spp.	*Daphnia pulicaria*
Shahmohamadloo et al. 2020 [24]	Median lethal concentrations in *C. dubia* (LC_50_ = 5.53 µg L^−1^) and *D. magna* (LC_50_ = 85.72 µg L^−1^)	Canadian laboratory study	*Microcystis aeruginosa* CPCC 300	*Daphnia magna*; *Ceriodaphnia dubia*
Pawlik-Skowrońska et al. 2013 [45]	22.2 mg L^−1^ of microcystin and 14.4 mg L^−1^ anatoxins in the cyanobacteria	Zemborzycki Dam Reservoir in Lublin, Poland	*Anabaena planctonica*; *Anabena affinis*; *Microcystis* spp.	Bioaccumulation in *Abramis brama*
Li et al. 2010 [46]	The highest dissolved cyanotoxin concentrations: MC-RR 1.56 mg L^−1^, MCYR 0.066 mg L-1, MC-LR 0.544 mg L^−1^, and anatoxin-a 0.106 mg L^−1^; intracellular: MC-RR 70.1 mg L^−1^, MC-YR 3.76 mg L^−1^, MC-LR 24.6 mg L^−1^, and anatoxin-a 0.184 mg L^−1^.	Yanghe Reservoir, China	*Anabaena spiroides*	
Podduturi et al. 2021 [47]	240–985 ng L^−1^	Different waterbodies in Denmark	*Aphanizomenon*, *Cuspidothrix*, *Dolichospermum*, *Phormidium, Planktolyngbya*, *Synechocystis*.	
Vo et al. 2020 [42]	24 h LC_50_: MC-LR 247–299 µg L^−1^ Microcystins containing extract 331–409 µg L^−1^	Vietnam	*Microcystis aeruginosa*; *Cylindrospermopsis curvispora*	*Daphnia lumholtzi*
Krztoń et al. 2017 [48]	Microcystins (0.246 µg L^−1^, microcystin LR)	Vistula River Poland	*Microcystis ichthyoblabe*, *Microcystis wesenbergii*, *Woronichinia naegeliana*, *Dolichospermum* *planctonicum*, *Dolichospermum* *spiroides*, *Oscillatoria tenuis*, *Cuspidothrix issatschenkoi*, *Anabaena* spp.	19 species of rotifers, 14 cladoceran species, and 9 species of copepods

**Table 4:** Population growth rates of zooplankton cultured on cyanobacterial diets.

Author(s)	Zooplankton Taxa	Test Conditions	
		Control *Ankistrodesmus falcatus*	Treatment *Cylindrospermopsis raciborskii* STX-producing strain
da Costa et al. 2013 [70]	*Daphnia gessneri*	0.266	0.289–0.313
	*Moina micrura*	0.584	0.431–0.490
	*Daphnia pulex*	0.309	0.146–0.296
			0.05–8.94
Bednarska et al. 2011 [71]	*Daphnia magna*	*Scenedesmus acutus*	0.58–0.78
		*Cylindrospermopsis raciborskii*	0.2–0.42
Soares et al. 2010 [6]	*Brachionus calyciflorus*	*Scenedesmus*	0.2–0.8
		*Cylindrospermopsis*	0.1–0.4
		*Microcystis*	All dead
Hulot et al. 2012 [72]	*Daphnia magna*	*Planktothrix agardhii* MC or MC-free strains	1.28–1.62
Luo et al. 2015 [73]	*Daphnia similoides*	9 d decayed *Microcystis*	0.28
		*Scenedesmus obliquus*	0.35
Whittington and Walsh 2015 [74]	*Daphnia lumholtzi*	*S. obliquus*	1.98
		*Cylindrospermopsis*	1.25–1.6
Lamei et al. 2020 [75]	*Daphnia sinensis*	*Chlorella* alone	0.35
		*Chlorella pyrenoidosa* + *M. aeruginosa*-FACHB469 (F469) or *C. raciborskii* N8 (N8) 50% each	0.20–0.25
Sarma et al. 2019 [60]	*Brachionus havanaensis*	*Nannochloropsis*	0.403
		*Microcystis aeruginosa*	0.174
	*Brachionus angularis*	*Nannochloropsis*	0.302
		*Microcystis aeruginosa*	−0.076
Haney and Lampert 2013 [76]	*Daphnia galeata*	*Microcystis*	0.25
	*Daphnia carinata*		−0.1
	*Daphnia pulex (Arctic)*		0.05
Jiang et al. 2013 [77]	*Daphnia carinata*	*Chlorella pyrenoidosa* (Cp)	0.2
		25% Cp + 75% *Microcystis*	0.25
Zamora Barrios et al. 2017 [38]	*Brachionus calyciflorus*	Controls: 0.16–0.24	1.74–0.48 in crude extracts Mostly *Planktothrix, Anabaenopsis, Spirulina,* and *Microcystis*

## Data Availability

Data will be made available on reasonable request.
